# Use of Open-Source Epidemic Intelligence for Infectious Disease Outbreaks, Ukraine, 2022

**DOI:** 10.3201/eid3009.240082

**Published:** 2024-09

**Authors:** Anjali Kannan, Rosalie Chen, Zubair Akhtar, Braidy Sutton, Ashley Quigley, Margaret J. Morris, C. Raina MacIntyre

**Affiliations:** University of New South Wales, Kensington, Sydney, New South Wales, Australia

**Keywords:** artificial intelligence, open source, epidemics, outbreaks, Ukraine, bacteria, viruses, zoonoses

## Abstract

Formal infectious disease surveillance in Ukraine has been disrupted by Russia’s 2022 invasion, leading to challenges with tracking and containing epidemics. To analyze the effects of the war on infectious disease epidemiology, we used open-source data from EPIWATCH, an artificial intelligence early-warning system. We analyzed patterns of infectious diseases and syndromes before (November 1, 2021–February 23, 2022) and during (February 24–July 31, 2022) the conflict. We compared case numbers for the most frequently reported diseases with numbers from formal sources and found increases in overall infectious disease reports and in case numbers of cholera, botulism, tuberculosis, HIV/AIDS, rabies, and salmonellosis during compared with before the invasion. During the conflict, although open-source intelligence captured case numbers for epidemics, such data (except for diphtheria) were unavailable/underestimated by formal surveillance. In the absence of formal surveillance during military conflicts, open-source data provide epidemic intelligence useful for infectious disease control.

On February 24, 2022, Russia forces launched an armed attack against Ukraine (*1*), escalating the ongoing Russo-Ukrainian conflict that began in 2014 when Russia annexed Crimea and resulting in one of Europe’s biggest threats to peace and security since the Cold War (*2*). The healthcare sector in Ukraine has been heavily affected, and 18 months into the recent conflict, ≈10,000 civilians had died (*3*). Such conflict situations increase the risk for epidemics (*4*), and the disruption or cessation of public health surveillance creates challenges for tracking them. Rapid epidemic intelligence using open-source data may be an alternative form of surveillance for infectious disease outbreaks during times of conflict.

Before 2022, the healthcare system in Ukraine had already experienced major stressors, including 8 years of conflict in the eastern part of the country, followed by the COVID-19 pandemic (*1*). Several weeks before Russia invaded, the fourth COVID-19 wave in Ukraine peaked, further decreasing numbers of available healthcare staff and increasing stress on hospitals (*1*). As of February 20, 2022, only 34.5% of the Ukraine population had received 2 doses of COVID-19 vaccine and only 2% of the eligible population had received a booster (*5*).

Other vaccine-preventable diseases were also highly prevalent in Ukraine before the invasion, and some of the lowest vaccine coverage rates in Europe were in Ukraine (*5*). For example, vaccine-derived poliomyelitis reemerged in 2021, after previous cases in 2015 and 2016 (*6*,*7*). A polio vaccination campaign targeting 140,000 children was implemented shortly after the outbreak but was paused as the conflict began (*6*). Similarly, a large measles epidemic affected Ukraine during 2017–2019, in part because of low vaccination coverage, which was the lowest in Europe in 2016 (31%) (*5*). Although reported cases decreased substantially in 2020 (264 cases) and 2021 (16 cases), recent shortages in measles vaccines pose a threat (*8*).

Compared with rates for most other countries in Europe, rates of tuberculosis (TB) in Ukraine are higher, and a substantial proportion of infections are multidrug resistant (*5*). Before the recent conflict, the COVID-19 pandemic had also significantly affected TB diagnosis and treatment centers, thus hindering TB control (*9*).

In 2019, the second largest HIV/AIDS epidemic in eastern Europe and central Asia was in Ukraine; ≈1.0% of the Ukraine population were reported to be living with the infection (*10*,*11*). Drivers of the epidemic included risky drug injection practices and disruption of treatment centers because of conflict, which could further exacerbate the HIV/AIDS burden in Ukraine (*6*).

The ongoing conflict, escalated by the invasion, has resulted in the destruction of healthcare infrastructure in Ukraine (*12*). In the 11 months after Russia invaded, 707 attacks on the Ukraine healthcare system were reported (*12*). Disruption of vaccination programs, limited testing capacity, displacement, and overcrowding can increase the risk for reemergence of vaccine-preventable infections such as polio, COVID-19, influenza, measles, TB, and pertussis (*6*). In addition, water supplies in cities such as Mariupol are not safe to drink because of damaged sewage systems and raw sewage leakage into nearby rivers and streams, yet many people still drink contaminated water (*4*). Risk for various infectious diseases is further increased by lack of regular housing and shelter, reduced caloric intake, and poor hygiene and sanitation. 

After February 2022, formal surveillance for most infectious diseases ceased, and reporting of notifiable infectious diseases has since been minimal. Open-source data can help overcome the lack of formal disease surveillance by harnessing information from open sources such as news media, medical organizations, and social media. Use of artificial intelligence (AI) can generate early warning signals from open-source data and provide epidemiologic information regarding infectious diseases in the absence of formal surveillance in a war zone (*13*).

To analyze patterns of infectious diseases and syndromes before (November 1, 2021–February 23, 2022) and during (February 24–July 31, 2022) the invasion (hereafter called the conflict), we used EPIWATCH (https://www.epiwatch.org), an open-source AI-based epidemic early warning system that has been operating since 2016. EPIWATCH collects open-source data, which are then processed by 2 AI systems to generate epidemic signals (*13*); the system has the proven capacity to capture early warning signals for infectious diseases (*14*–*16*). Because our study involved analysis of open-source published data that was anonymous, ethics approval was not required.

## Methods

To enable comparison of periods before and during the conflict, we gathered open-source data from EPIWATCH for Ukraine for 2 periods before (November 1, 2021–February 23, 2022, and February–July 2021) and during (February 24–July 31, 2022 the conflict. EPIWATCH collects outbreak reports on specific infectious diseases and clinical syndromes, including undiagnosed syndromes such as severe acute respiratory infection, pneumonia, rash and fever, and encephalitis.

The before-conflict period served as a baseline for comparing disease reports from the during-conflict period. To capture a relevant snapshot of epidemics in Ukraine around the time the conflict began, which could influence epidemics occurring during the conflict, we selected the 3 months before the conflict as a baseline for the immediate before-conflict period. To account for seasonal variations of disease incidence, we also collected data for the same period during the previous year (February 24–July 31, 2021) as a second comparator.

EPIWATCH searches for ≈200 specific disease and syndrome terms in 46 languages, and ≈70% of all intelligence gathered is from non-English sources (*17*). EPIWATCH gathered data for all areas within the internationally recognized borders of Ukraine in the Ukrainian and Russian languages (because Russian is spoken in some parts of Ukraine). The Russian language had already been in use in EPIWATCH since September 2019. After Russia invaded Ukraine on February 24, 2022, the EPIWATCH team added the Ukrainian language to the EPIWATCH search engine. To reduce ascertainment bias caused by adding a new language, identical EPIWATCH search terms were applied retrospectively in the Ukrainian language by using the Google search engine for the 2 before-conflict control periods in 2021–2022; those terms were added to the dataset to compare trends before and during the conflict. Because the retrospective search used the same search terms and search engine (Google) for prospective data collection, the results should be comparable.

We downloaded EPIWATCH outbreak report data for Ukraine for the periods of interest, combined with retrospective data in the Ukrainian language, and screened the data for eligibility. Inclusion criteria for the final analysis were reports focusing on infectious diseases and syndromes among humans; zoonotic diseases (diseases circulating among animals that can be transmitted to humans); and having data regarding confirmed, probable, or suspected cases. We excluded reports not meeting those criteria.

To conduct descriptive epidemiologic analysis of outbreak reports, we used Microsoft Excel (https://www.microsoft.com). We extracted data on diseases, syndromes, populations affected, and location and time and analyzed the data by reporting periods. We compared numbers of reports from the before- and during-conflict periods as well as from the previous year and constructed graphs by using Prism (GraphPad, https://www.graphpad.com).

We next extracted case numbers from the reports. We extracted from EPIWATCH reports the number of cases of the 8 most reported diseases and created a line list for further analysis. We included cases in the line list only if reports had definitive numbers. If case numbers were vague or cumulative, we excluded them. We also removed duplicate case numbers. Then, we searched for formal case numbers published by organizations engaged in formal surveillance (e.g., the World Health Organization and the European Centre for Disease Control and Prevention). We then compared during-conflict case numbers from EPIWATCH with case numbers of the same diseases published by formal surveillance during the same period, when available.

## Results

**Figure 1:**
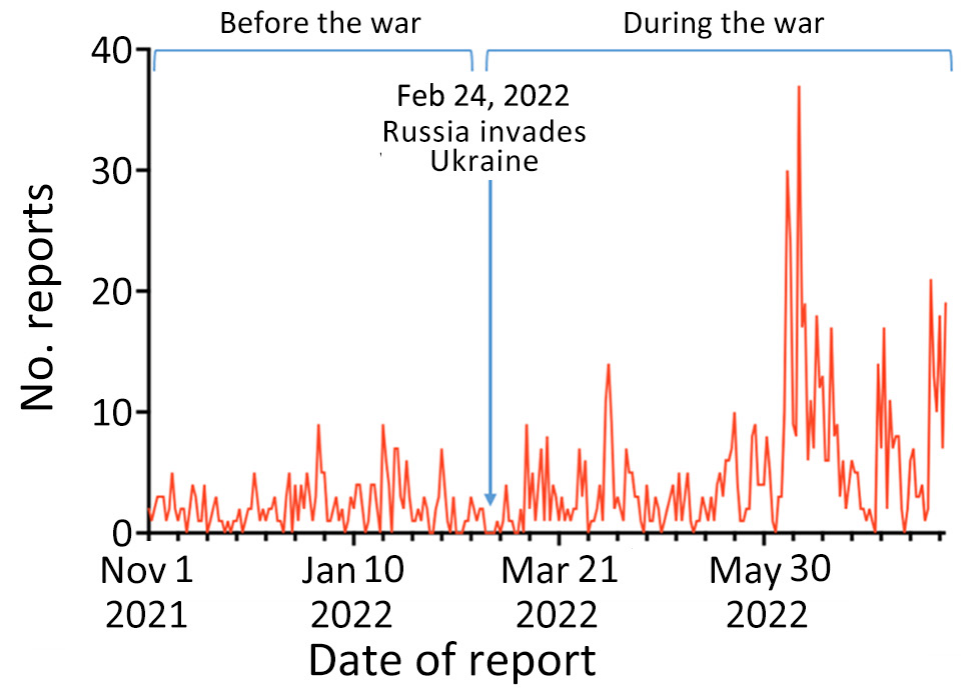
Daily EPIWATCH (https://www.epiwatch.org) reports of outbreaks in Ukraine before and during Russia’s invasion, November 1, 2021–July 31, 2022.

**Figure 2:**
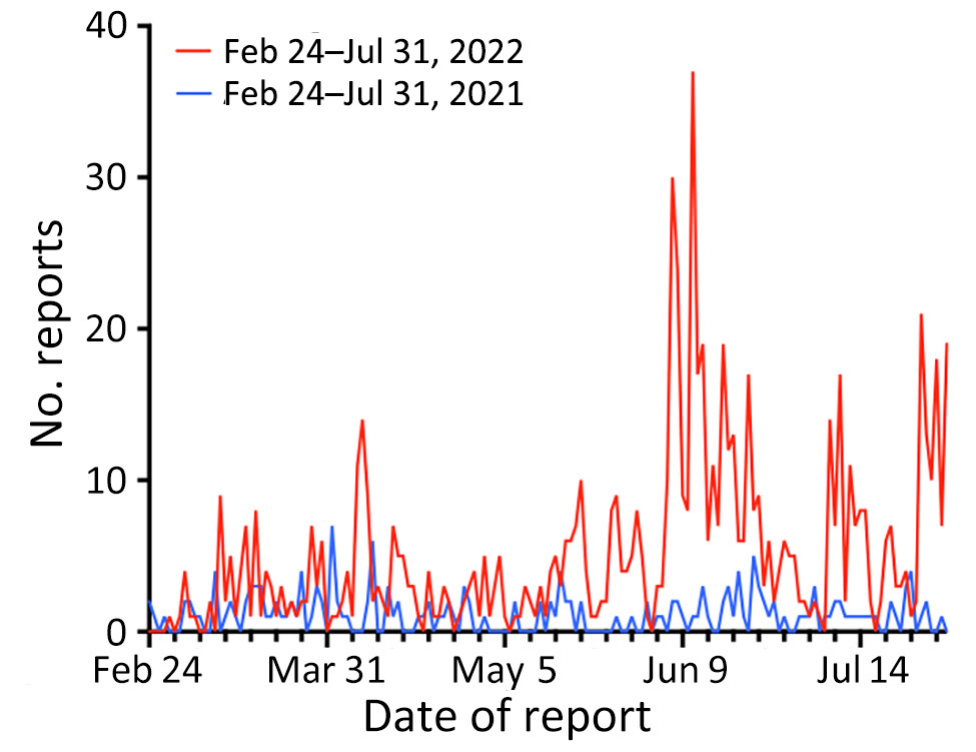
Comparison of daily EPIWATCH (https://www.epiwatch.org) reports of outbreaks in Ukraine during Russia’s invasion (February 24, 2022–July 31, 2022) and in the same period of the previous year (February 24, 2021–July 31, 2021).

We identified 805 outbreak reports in EPIWATCH during February 24, 2022–July 31, 2022, for an average of 5 reports per day. In comparison, 259 reports were collected in the 3 months before the conflict (November 1, 2021–February 23, 2022), an average of 2 reports per day (Figure 1). Reports initially peaked (n = 14) on April 7, 2022, largely regarding a diphtheria outbreak in the Ternopil Oblast of western Ukraine. The largest peak was in June, when 37 reports were attributed to an outbreak of cholera in Mariupol in eastern Ukraine. For the same period in the previous year, we found 180 reports, indicating a 447% increase during the conflict (Figure 2).

**Figure 3:**
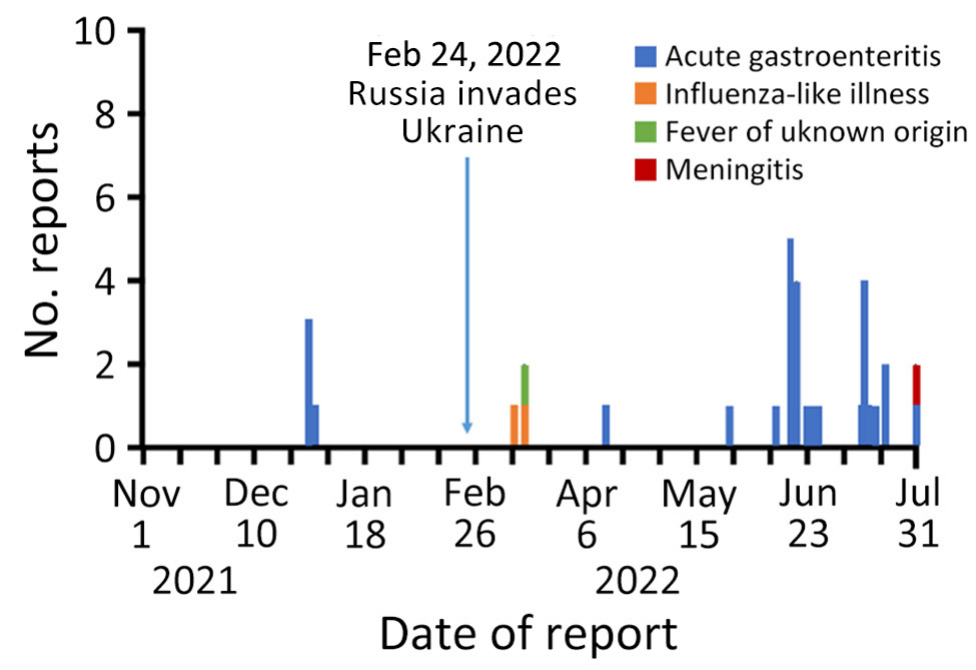
Number of reports of clinical syndromes per day before and during Russia’s invasion, Ukraine, November 1, 2021—July 31, 2022.

Before the invasion, there were a total of 4 reports of clinical syndromes, all of which were acute gastroenteritis (Figure 3). After the invasion, syndromic reports increased; acute gastroenteritis (87.1% of reports) was the most common, followed by influenza-like illness (6.5%), fever of unknown origin (3.2%), and meningitis (3.2%).

According to individual reports from June 2022 of acute gastroenteritis, many illnesses were identified as dysentery or suspected cholera. Among other reports of acute gastroenteritis, a report from May 26, 2022, cited an outbreak of unknown cause resulting in acute gastroenteritis signs/symptoms among 17 internally displaced persons in the Lviv region. In addition, starting on July 20, 2022, an outbreak affecting 91 persons within 1 week was reported in the Odessa region. A report of meningitis recorded on July 31, 2022, mentioned an overall increase in viral/bacterial meningitis cases in Ukraine since before the conflict.

Before the conflict, the most frequently reported outbreaks were COVID-19, influenza, and poliomyelitis, accounting for 70% of the total reports during that period (Table 1). In the during-conflict period, the most frequently reported diseases were COVID-19 (increased from 106 to 187 reports), cholera (from 0 to 157 reports), botulism (from 22 to 122 reports), TB (from 11 to 75 reports), HIV/AIDS (from 6 to 61 reports), rabies (from 12 to 40 reports), salmonellosis (from 4 to 39 reports), and diphtheria (from 2 to 29 reports) (Table 2). Only cholera was not reported before the conflict. Frequency of reporting decreased for poliomyelitis (from 37 to 19 reports), influenza-unspecified (from 39 to 10 reports), and influenza A(H3N2) (0 reports) during the conflict (Table 1). 

**Table 1:** Numbers of daily reports of disease outbreaks before and during Russia’s invasion, by disease, Ukraine, November 1, 2021–July 31, 2022*

Disease	No. reports	
	Before conflict	During conflict
COVID-19	106	187
Cholera	0	157
Botulism	22	122
Tuberculosis	11	75
HIV/AIDS	6	61
Rabies	12	40
Salmonellosis	4	39
Diphtheria	2	29
Poliomyelitis	37	19
Varicella	1	15
Rotavirus infection	9	15
Malaria	8	13
Influenza	39	10
Measles	1	9
Leptospirosis	1	8
SARS-CoV-1 infection	3	8
Other†	2	7
Hepatitis A	0	6
*Escherichia coli* infection	2	5
Hepatitis, unspecified	0	4
Hepatitis C	1	4
Lyme disease	0	3
Encephalitis	0	3
Tularemia	1	3
*Staphylococcus* infection	2	3
Anthrax, unspecified	0	2
Mpox	0	2
Shigellosis	0	2
Smallpox	0	2
Meningococcal	2	2
CCHF	0	1
Newcastle disease	0	1
Q fever	0	1
Tetanus	0	1
Yersiniosis	0	1
Typhoid fever	1	1
Dirofilariasis	2	1
Avian influenza, unspecified	4	1
Brucellosis	1	0
Influenza A(H1N1)	3	0
Influenza A(H3N2)	11	0
Influenza A(H5N1)	1	0
Influenza B	1	0
Listeriosis	5	0
Pneumonia of unknown origin	1	0
Rubella	1	0
Trichinellosis	1	0

*Data are reports of outbreaks, not case numbers. CCHF, Crimean-Congo hemorrhagic fever. †Reports containing information about diseases that were deemed by EPIWATCH analysts to not fit in any of the existing disease/syndrome categories on EPIWATCH.

**Table 2:** Case numbers for the most frequently reported diseases seasonally and before and during Russia’s invasion, by period, Ukraine

Disease	Seasonal, February 24–July 31, 2021	Before invasion, November 1, 2021–February 23, 2022	During invasion, February 24–July 31, 2022
COVID-19	279,671	599,695	7,338
Cholera	0	0	10,015
Botulism	9	11	32
Tuberculosis	5,851	4,799	5,647
HIV + AIDS	3,717	3,255	4,333
Rabies			
Human	0	2	13
Animal	0	11	20
Salmonellosis	109	0	57
Diphtheria	0	7	2

With regard to our comparison of case numbers from EPIWATCH and from formal surveillance, most diseases were not reported by formal surveillance, except for botulism, TB, and diphtheria (Table 3). For botulism, the number of cases identified through EPIWATCH (32 cases) was lower than that identified through formal surveillance (51 cases) (*22*). For TB, the only statistic found by formal surveillance was for the Rivne Oblast for the first quarter of 2022 (113 cases), whereas EPIWATCH was able to collect case number data for the entire reporting period and across more locations (5,647 cases) (*23*). Both EPIWATCH and formal surveillance identified 2 cases of diphtheria (*22*).

**Table 3:** Comparison of cases extracted from outbreak reports collected by EPIWATCH and by formal surveillance during Russia’s invasion for the 8 most frequently reported diseases, Ukraine, February 24–July 31, 2022*

Disease	No. cases obtained from EpiWatch	No. cases reported by government or other surveillance
COVID-19	7,338	COVID-19 surveillance in Ukraine ceased in February 2022 (*18*).
Cholera	10,015	None. The WHO situation report from June 22, 2022, mentions increased social media publications about the threat of cholera, and the report from June 29, 2022, mentions the detection of non-O1 *Vibrio cholerae* environmental samples; however, no case numbers were reported (*19**,**20*). In December 2022, WHO published a 2022 global situational summary report on cholera; however, it did not mention of cases of cholera in Ukraine (*21*).
Botulism	32	51 cases were reported by the WHO Health Cluster Ukraine in the first 6 months of 2022 (*22*).
Tuberculosis	5,647	113 cases were reported by the WHO Health Cluster Ukraine in the Rivne Oblast for the first quarter of 2022, with a last update on April 2022 (*23*).
HIV + AIDS	4,333	None. The cessation of HIV/AIDS surveillance has not officially been announced; no government or other reporting was found.
Rabies		None. The cessation of rabies surveillance has not officially been announced; no government or other reporting was found.
Human	13	
Animal	20	
Salmonellosis	57	None. The cessation of salmonellosis surveillance has not officially been announced; no government or other reporting was found.
Diphtheria	2	2 cases were reported by the WHO Health Cluster Ukraine, with last update in April 2022 (*22*).

*EPIWATCH (https://www.epiwatch.org) statistics are for total cases in the entire country and not specific oblasts. WHO, World Health Organization.

## Discussion

The ongoing escalation of the conflict in Ukraine is one of the world’s fastest growing humanitarian crises and has disrupted health systems and reduced outbreak detection and response capabilities in the country (*24*). We have demonstrated the value of using open-source epidemic intelligence to gain information about unfolding epidemics and public health priorities in a conflict zone where formal surveillance is reduced or lacking. We were able to identify new epidemics that occurred during the conflict, such as cholera, botulism, and human cases of rabies (which were presumably exacerbated by an increased number of displaced domestic dogs). The increases in cholera and gastroenteritis reflect declining hygiene and sanitation during the conflict, including lack of access to safe drinking water and toilets and subsequent improper disposal of fecal waste (*25*). The increase may also be exacerbated by improper handling and disposal of dead bodies (*26*), such as burial in shallow graves, which increases the risk for transmission of some diseases (*26*,*27*). We also identified increased HIV/AIDS and TB cases. HIV clinics were shut down during the conflict (*4*), most likely affecting testing and surveillance. In addition, medications such as antiretroviral drugs have become scarce or been misused, which may increase the risk for drug-resistant HIV and may subsequently limit treatment options and further increase transmission (*6*). Overall, the disruption of transportation networks during the conflict has decreased access to medical supplies and treatment for infectious diseases (*4*). Lack of access to testing and treatments has also resulted in loss of continuity of care, poorer outcomes, and increased community transmission (*6*).

Using EPIWATCH, we were able to extract more complete case data for the 8 most reported infectious diseases compared with formal surveillance. During the conflict, formal reporting of infectious diseases such as COVID-19 notably decreased, most likely because of lack of testing. The collapse of formal surveillance systems in Ukraine during the conflict resulted from a variety of factors (e.g., high levels of displacement, attacks on healthcare facilities, lack of routine data collection, limited testing and treatment, reduced diagnostic capabilities, and changes in disease testing policies) (*28*). After the invasion, case numbers from the Ukraine government or other formal surveillance sources for many diseases either ceased to be reported or were not up to date. Some formal surveillance systems continued to report cases of botulism, TB, and diphtheria, but even for those, we showed that numbers are probably underestimated.

Weak or absent formal surveillance during wartime hampers timely and targeted interventions such as vaccination programs (*29*). Solely relying on formal surveillance may result in missed early warning signals from other sources, heightening the risk that diseases will spread internationally, particularly if refugees migrate to other countries. In that context, open-source epidemic intelligence can provide early warnings of epidemics.

Among the limitations of our study, open-source data are not subject to validation compared with formal surveillance data. However, EPIWATCH uses 3 AI subsystems to improve data quality and exclude irrelevant material (*30*). All data are then further curated by human analysts, who follow standardized operating procedures. EPIWATCH provides reports of outbreaks, rather than case numbers; thus, monitoring report trends reflects signal strength, and case numbers extracted from reports may be less precise. However, for most diseases there was no formal surveillance or case reporting during the conflict, whereas we were able to extract case numbers from the open-source reports. Another limitation is ascertainment bias because media reporting may be increased in regions of Ukraine with larger populations. As a result, data from smaller regions were less frequently obtained, and thus, infectious diseases may be underestimated. Last, because we added the Ukrainian language in February 2022, ascertainment bias could have contributed to the observed increase in reports from that time. However, we accounted for that difference by using a control period in the previous year, for which we retrospectively collected data in the Ukrainian language. Historical open-source reports of outbreaks from Ukraine might have been removed or censored by 2022, which may have reduced the potential for data capture; however, there is no evidence to suggest this was the case. A longer control period may have been more informative of longer-term trends and is planned in a follow-up study.

Our study provides an overview of epidemic activity in Ukraine during the Russian escalation of the Russo-Ukrainian war in 2022, demonstrating that EPIWATCH was able to capture a breadth of data that was not captured by other formal sources. Open-source digital disease surveillance therefore provides a useful way to gather real-time health intelligence in a conflict zone when formal surveillance is absent or reduced. Using EPIWATCH and other open-source health intelligence systems can be valuable for real-time decision support in disaster contexts, including conflict or natural disasters.
